# Numerical study of bio-convection flow of magneto-cross nanofluid containing gyrotactic microorganisms with activation energy

**DOI:** 10.1038/s41598-021-95587-2

**Published:** 2021-08-06

**Authors:** Qiu-Hong Shi, Aamir Hamid, M. Ijaz Khan, R. Naveen Kumar, R. J. Punith Gowda, B. C. Prasannakumara, Nehad Ali Shah, Sami Ullah Khan, Jae Dong Chung

**Affiliations:** 1 Department of Mathematics, Huzhou University, Huzhou, 313000 People’s Republic of China; 2 Department of Mathematics, Quaid-i-Azam University, Islamabad, 44000 Pakistan; 3 Department of Mathematics and Statistics, Riphah International University, I-14, Islamabad, 44000 Pakistan; 4 Department of Studies and Research in Mathematics, Davangere University, Davangere, Karnataka India; 5 Department of Mechanical Engineering, Sejong University, Seoul, 05006 Korea; 6 Department of Mathematics, Lahore Leads University, Lahore, Pakistan; 7 Department of Mathematics, COMSATS University Islamabad, Sahiwal, 57000 Pakistan

**Keywords:** Engineering, Mathematics and computing

## Abstract

In this study, a mathematical model is developed to scrutinize the transient magnetic flow of Cross nanoliquid past a stretching sheet with thermal radiation effects. Binary chemical reactions and heat source/sink effects along with convective boundary condition are also taken into the consideration. Appropriate similarity transformations are utilized to transform partial differential equations (PDE’s) into ordinary ones and then numerically tackled by shooting method. The impacts of different emerging parameters on the thermal, concentration, velocity, and micro-rotation profiles are incorporated and discussed in detail by means of graphs. Results reveal that, the escalation in magnetic parameter and Rayleigh number slowdowns the velocity and momentum of the fluid. The increase in Biot number, radiation and heat sink/source parameters upsurges the thermal boundary but, converse trend is seen for escalating Prandtl number. The density number of motile microorganisms acts as a growing function of bioconvection Lewis number and declining function of bioconvection Peclet number.

## Introduction

Researchers and scientists nowadays have more research fields due to developments in nanotechnology and nanoscience. Nanofluids are proving to be useful in a variety of situations, including heat transference, technological advances demand efficient heat conveyance processes, and nanoliquids provide a more effective medium for heat passage from one source to the other. In this regard different fluid models have been presented over time to illustrate fluid properties. The Cross-viscosity model for generalized Newtonian fluid was proposed by Cross. By using this concept, Khan et al.^1^ elucidated the chemically reacting magnetohydrodynamic (MHD) radiative flow of cross nanoliquid towards a stretchy sheet. Ali et al.^2^ explicated the radiative stream of cross nanoliquid instigated by a stretchy sheet. Abbas et al.^3^ expounded the MHD convective stream of cross nanoliquid with thermal radiation. Xiong et al.^4^ illuminated the chemically reacting dissipative flow of cross nanoliquid with mixed convection. Hamid et al.^5^ investigated the axisymmetric stream of cross nanoliquid flow with radiation and chemical reaction effect.

The studies of magnetohydrodynamic (MHD) streams of non-Newtonian and Newtonian liquids over a stretching surface have gotten a lot of interest because of their wide range of uses in chemical engineering and metallurgy. Such MHD flow investigations are critical in industry and have uses in a number of fields, including petroleum processing and metallurgical processes. The rate of cooling, as well as the desired end product properties, can be regulated by using electrically conducting fluids and applying a magnetic field. In the purification of molten metals from nonmetallic inclusions, a magnetic field has been used. Recently, Hayat et al.^6^ elucidated the MHD radiative stream of liquid past a porous stretchy sheet with thermal radiation. Senapati et al.^7^ expounded the MHD flow of Casson nanoliquid flow above a stretch sheet. Patil et al.^8^ deliberated the chemically reacting radiative stream of Powel–Eyring liquid above a stretchy sheet. Uddinet al.^9^ elucidated the MHD radiative stream of Prandtl–Eyring nanoliquid above a stretchy sheet with mixed convection. Nayak et al.^10^ explicated the MHD steam of micropolar Casson cross nanoliquid.

The mechanism of thermal radiation involves a hot body releasing electromagnetic radiation in both directions. Many objects on the planet emit radiation in the infrared range of the electromagnetic spectrum. Thermal radiation is important in heat nuclear reactor protection, power plants, exchangers, furnace architecture, solar energy, and power technology. Motivated by these uses, Khan et al.^11^ explored the radiative flow of Carreau liquid above a stretchy surface. Hashim et al.^12^ explicated the radiative stream of Williamson nanoliquid above a stretchy geometry. Ali et al.^13^ elucidated the radiative flow of nanoliquid with heat production/absorption. Reddy et al.^14^ expounded the radiative flow of nanoliquid past a melting surface. Xionget al.^15^ explicated the effect of radiation and heat source/sink on flow of hybrid nanoliquid.

The relationships between chemical reactions and mass transport are typically complex, as shown by the synthesis and absorption of reactant species at various rates both within the liquid and during mass transfer. Chemical reactions have abundant usages in fields of engineering and sciences like electric power generator and food processing. The concept of binary chemical reaction was initially elucidated by Merkin^16^. Rasool et al.^17^ exemplified the impact of binary chemical reaction on Williamson nanoliquid above anelastic sheet. Wang et al.^18^ elucidated the chemically reactive dissipative stream of Carreau nanoliquid above a stretchy surface. Gowda et al.^19^ expounded the impact of chemical reaction on nanoliquid stream past a stretchy sheet coiled in a circle. Khan and Alzahrani^20^ expounded the impact of binary chemical reaction on radiative stream of Walter-B nanoliquid.

Bio-convection is a natural process that occurs as microorganisms move randomly in single-celled or colony-like formation. The directional motion of various forms of microorganisms is the basis for various bio-convection systems. Gyrotactic microorganisms are those that swim upstream against gravity in still water, causing the upper portion of the suspension to be denser than the lower part. Bioconvection's importance can be seen in a variety of bio-microsystems, such as biotechnology related to mass transportation, enzyme biosensors and mixing. Recently, Khan et al.^21^ elucidated the MHD bioconvective stream of Newtonian fluid with chemical reaction. Chu et al.^22^ explicated the MHD bioconvective stream of third grade liquid past a stretchy sheet by using Buongiorno model. Al-Khaled et al.^23^ expounded the radiative bioconvective stream of nanoliquid. Zadeh et al.^24^ elucidated the bioconvectionflow of micropolar liquid instigated by anupright sheet. Waqas et al.^25^ explicated bioconvective stream of second-grade nanofluid with radiation effect.

It is familiar that there are various approaches that could be contemplated in order to explain few realistic solutions for this specific type of issue. However, to the best of the authors' understanding, no numerical solution has been earlier inspected for magnetic flow of Cross nanofluid past a stretching sheet with thermal radiation effects. Also, the findings of this research are completely new and have never been published previously. The focal point in the current paper is to examine the above-described flow numerically. Our findings from this research are likely to provide not only useful information for applications, but also a supplement to the current literature. These impacts will be unique and novel, according to the most effective of our data.

## Mathematical formulation

**Figure 1:**
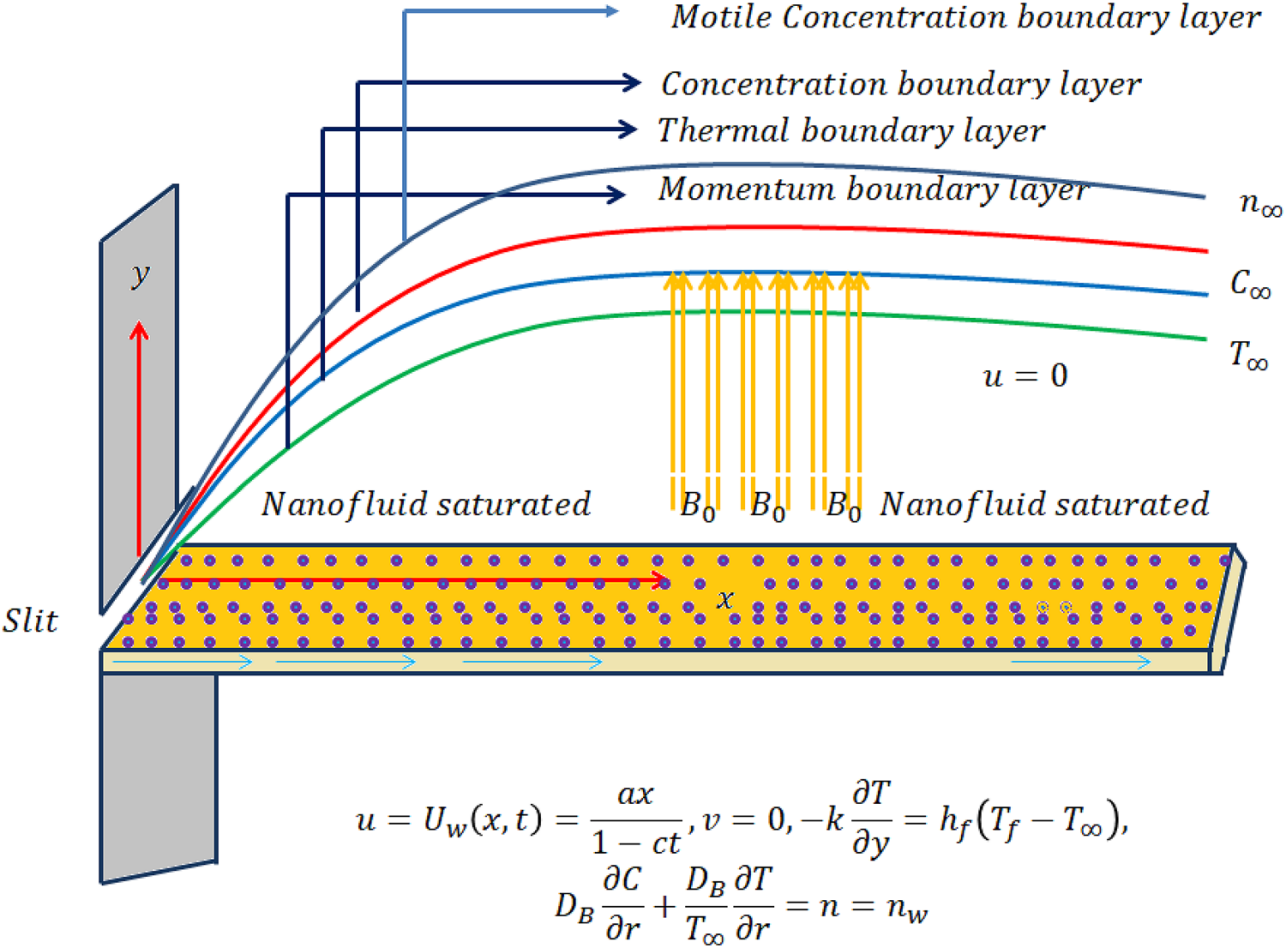
Schematic sketch of the physical model.

In this study, we have considered transient two-dimensional magnetic flow of an incompressible Cross nanofluid past a stretching sheet in the presence of thermal radiation effects. A non-uniform time dependent transverse magnetic field of strength $B_{0}$ is applied perpendicular to flow direction (see Fig. 1^26^). Since the magnetic Reynolds number is low, the induced magnetic field can be ignored. The effect of Brownian motion and thermophoresis is incorporated into a currently developed framework for nanofluid. The implementation of a critical practical concentration condition, namely the zero nanoparticles mass flux condition and also, the convective boundary condition are taken into consideration. Binary chemical reactions and heat source/sink effects are also taken into consideration. The suspension of nanoparticles in nanofluid is achieved through the use of a surfactant or surface charge technology. This prevents nanoparticles from adhering to the surface and accumulating.

The governing equations of the above-described flow are^1–4^:

*Continuity:*

1 $$ \frac{\partial u}{{\partial x}} + \frac{\partial u}{{\partial y}} = 0. $$

*Momentum equation*:

2 $$ \frac{{{\mathbf{\partial }}u}}{{{\mathbf{\partial }}t}} + u\frac{{{\mathbf{\partial }}u}}{{{\mathbf{\partial }}x}} + v\frac{{{\mathbf{\partial }}u}}{{{\mathbf{\partial }}y}} = \nu \left[ {\frac{{\frac{{{\mathbf{\partial }}u}}{{{\mathbf{\partial }}y}}}}{{1 + \left\{ {\Gamma \left( {\tfrac{\partial u}{{\partial y}}} \right)^{1 - n} } \right\}^{2} }}} \right] - \frac{{\sigma B^{2} (t)}}{{\rho_{f} }}u + \frac{1}{{\rho_{f} }}\left[ \begin{gathered} g\rho_{f} (T - T_{{_{\infty } }} )\beta (1 - C_{{_{\infty } }} ) \hfill \\ - g(\rho_{p} - \rho_{f} )(C - C_{{_{\infty } }} ) \hfill \\ - g(\rho_{m} - \rho_{\infty } )\gamma (n - n_{{_{\infty } }} ) \hfill \\ \end{gathered} \right]. $$

*Energy equation*:

3 $$ \frac{{{\mathbf{\partial }}T}}{{{\mathbf{\partial }}t}} + u\frac{\partial T}{{\partial x}} + v\frac{\partial T}{{\partial y}} = \left[ {\alpha_{f} + \frac{{16\sigma^{ * } T_{{_{\infty } }}^{3} }}{{3k^{ * } \left( {\rho c_{f} } \right)}}\frac{{\partial^{2} T}}{{\partial y^{2} }}} \right] + \tau \left[ {D_{B} \frac{\partial C}{{\partial r}}\frac{\partial T}{{\partial r}} + \frac{{D_{T} }}{{T_{\infty } }}\left( {\frac{\partial T}{{\partial r}}} \right)^{2} } \right] + \frac{{Q_{0} }}{{\left( {\rho c_{p} } \right)_{f} }}\left[ {(T - T_{{_{\infty } }} )} \right]. $$

*Concentration equation*:

4 $$ \frac{{{\mathbf{\partial }}C}}{{{\mathbf{\partial }}t}} + u\frac{\partial C}{{\partial x}} + v\frac{\partial C}{{\partial y}} = D_{B} \frac{{\partial^{2} C}}{{\partial y^{2} }} + \frac{{D_{T} }}{{T_{\infty } }}\left( {\frac{{\partial^{2} C}}{{\partial y^{2} }}} \right) - k_{r}^{2} (C - C_{\infty } )\left( {\frac{T}{{T_{\infty } }}} \right)^{{n^{ * } }} \exp \left( { - \frac{{E_{a} }}{{k^{ * } T}}} \right). $$

*Bioconvection equation*:

5 $$ \frac{{{\mathbf{\partial }}n}}{{{\mathbf{\partial }}t}} + u\frac{\partial n}{{\partial x}} + v\frac{\partial n}{{\partial y}} + \frac{{b_{1} W_{c} }}{{C_{\infty } }}\left[ {\frac{\partial }{\partial y}\left( {n\frac{\partial C}{{\partial r}}} \right)} \right] = D_{m} \left( {\frac{{\partial^{2} n}}{{\partial y^{2} }}} \right). $$

The physical realistic boundary conditions are

6 $$ u = U_{w} \left( {x,\,t} \right) = \frac{ax}{{1 - ct}},\,\;v = 0,\, \, - k\frac{\partial T}{{\partial y}} = h_{f} \left( {T_{f} - T_{\infty } } \right),\, \, D_{B} \frac{\partial C}{{\partial r}} + \frac{{D_{T} }}{{T_{\infty } }}\frac{\partial T}{{\partial r}} = 0,\,n = n_{w} {\text{ at }}y = 0, $$

7 $$ u \to 0,\, \, T \to T_{\infty } ,\, \, C \to C_{\infty } ,\,n \to n_{\infty } {\text{ as }}y \to \infty . $$

We have the similarity transformations

8 $$ \eta = y\sqrt {\frac{{U_{w} }}{\nu x}} ,\, \, \psi = \sqrt {\nu U_{w} x} f\left( \eta \right),\, \, \theta \left( \eta \right) = \frac{{T - T_{\infty } }}{{T_{f} - T_{\infty } }},\, \, \phi \left( \eta \right) = \frac{{C - C_{\infty } }}{{C_{\infty } }},\,\chi \left( \eta \right) = \frac{{n - n_{\infty } }}{{n_{w} - n_{\infty } }}. $$

The stream function $\psi (r,\,x)$ is given by $u = \tfrac{1}{r}\tfrac{\partial \psi }{{\partial r}}$ and $v = - \tfrac{1}{r}\tfrac{\partial \psi }{{\partial x}},$

By utilizing Eq. (8) in Eqs. (1)–(5) with associated boundary constraints revealed in Eqs. (6) and (7), we came to know that Eq. (1) is satisfied and Eqs. (2–5) take the following form

9 $$ \begin{aligned} & \left( {ff^{\prime\prime} - (f^{\prime})^{2} } \right)[1 + \left( {Wef^{\prime\prime}} \right)^{1 - n} ] - M^{2} f^{{^{\prime } }} \left[ {1 + \left( {Wef^{\prime\prime}} \right)^{1 - n} } \right] + [1 + n\left( {Wef^{\prime\prime}} \right)^{1 - n} ]f^{\prime\prime\prime} \\ & \quad - A\left( {f^{\prime} + \frac{\eta }{2}f^{\prime\prime}} \right) + \lambda (\theta - N_{r} \phi - R_{b} \chi ) = 0, \\ \end{aligned} $$

10 $$ \left( {1 + \frac{4}{3}Rd} \right)\frac{1}{\Pr }\theta^{\prime\prime} + f\theta^{\prime} + A\frac{\eta }{2}\theta^{\prime} + N_{b} \theta^{\prime}\phi^{\prime} + N_{t} \theta^{\prime 2} + Q_{0} \theta = 0, $$

11 $$ \phi^{\prime\prime} + Scf\phi^{\prime} + \frac{{N_{t} }}{{N_{b} }}\theta^{\prime\prime} + ScA\frac{\eta }{2}\phi^{\prime} - \sigma (1 + \delta \theta )^{{n^{ * } }} \phi \exp \left( { - \frac{{E_{1} }}{1 + \delta \theta }} \right) = 0, $$

12 $$ \chi^{\prime\prime} + Lbf\chi^{\prime} - Pe\left[ {\chi^{\prime}\phi^{\prime} + \left( {\chi + \Omega } \right)} \right] + LbA\frac{\eta }{2}\chi^{\prime} = 0, $$

and transformed boundary conditions are:

13 $$ f(0) = 0,\, \, f^{\prime}(0) = 1,\, \, \theta^{\prime}(\eta ) = - Bi\left[ {1 + \theta (\eta )} \right],\, \, N_{b} \phi^{\prime} + N_{t} \theta^{\prime} = 0,\chi \left( 0 \right) = 1, $$

14 $$ f^{\prime}(\infty ) \to 0,\, \, \theta (\infty ) \to 0,\, \, \phi (\infty ) \to 0,\,\chi (\infty ) \to 0, $$

where, prime denotes the derivative with respect to $\eta$ and the dimensionless physical parameters are defined as follows:

$M = \tfrac{{\sigma B_{0}^{2} }}{2\rho a}$ is the magnetic parameter, $Lb = \left( {\tfrac{\alpha }{{D_{n} }}} \right)$ is bioconvection Lewis number, $Rd = \tfrac{{4\sigma^{ * } T_{\infty }^{3} }}{{3k^{ * } k_{f} }}$ denotes the radiation parameter, $We = a\Gamma Re^{1/2}$ is the Weissenberg number, $\;Q_{0} = \left( {\tfrac{Q(1 - ct)}{{a(\rho c)_{f} }}} \right)$ heat generation/absorption parameter, $Nb = \left( {\tfrac{{\tau D_{B} (C_{f} - C_{\infty } )}}{\nu }} \right)$ Brownian motion parameter, $A = \left( {\tfrac{c}{a}} \right),$ unsteadiness parameter, $Sc = \left( {\tfrac{\nu }{{D_{B} }}} \right)$ the Schmidt number, $\Pr = \left( {\tfrac{{\mu c_{p} }}{k}} \right),$ Prandtl number, $Pe = \left( {\tfrac{{b_{1} W_{c} }}{{D_{n} }}} \right)$ the bioconvection Peclet number, $Le = \left( {\tfrac{\alpha }{{D_{B} }}} \right)$ the traditional Lewis number, $Nt = \left( {\tfrac{{\tau D_{B} (T_{f} - T_{\infty } )}}{{\nu T_{\infty } }}} \right)$ the thermophoresis parameter, $N_{r} = \left( {\tfrac{{(\rho_{p} - \rho_{f} )\Delta C_{w} }}{{\rho_{f} \beta (1 - C_{{_{\infty } }} )\Delta T_{f} }}} \right)$ the buoyancy ratio parameter, $Rb = \left( {\tfrac{{\gamma \Delta \rho \Delta n_{w} }}{{\rho_{f} \beta (1 - C_{{_{\infty } }} )\Delta T_{f} }}} \right)$ the bioconvection Rayleigh number, $\sigma = \left( {\tfrac{{n_{\infty } }}{{\Delta n_{w} }}} \right)$ the bioconvection constant.

It should be remembered that the physical quantities of engineering concern, namely the local skin friction coefficient, local Nusselt quantity, and motile microorganisms, are also essential characteristics of the current investigation.

15 $$ C_{f} = \frac{{\tau_{w} }}{{\rho U_{w}^{2} }},\,Nu_{x} = \frac{{xq_{w} }}{{k_{f} \left( {T_{f} - T_{\infty } } \right)}},\,Nn_{x} = \frac{{xq_{n} }}{{D_{n} \left( {n_{w} - n_{\infty } } \right)}}, $$

where $\tau_{w}$, $q_{w},$ $q_{n}$ is the wall shear stress and wall heat flux and wall motile microorganism flux, respectively, having the following form:

16 $$ \tau_{w} = \mu_{0} \left( {\frac{{\tfrac{{{\mathbf{\partial }}u}}{{{\mathbf{\partial }}y}}}}{{1 + \left\{ {\Gamma \left( {\tfrac{\partial u}{{\partial y}}} \right)^{1 - n} } \right\}}}} \right)_{y = 0} ,\, \, q_{w} = - k\left( {\frac{\partial T}{{\partial y}}} \right)_{y = 0} ,\,q_{n} = - D_{n} \left( {\frac{\partial n}{{\partial y}}} \right)_{y = 0} . $$

Following relations in dimensionless form are:

17 $$ Re^{1/2} C_{f} = \frac{{2f^{\prime\prime}(0)}}{{[1 + \left\{ {Wef^{\prime\prime}(0)} \right\}^{1 - n} ]}},\,\,Re^{ - 1/2} Nu_{x} = - \left( {1 + \frac{4}{3}Rd} \right)\,\theta^{\prime}(0),\,\,Re^{ - 1/2} Nn_{x} = - \chi^{\prime}(0). $$

## Numerical scheme

The numerical simulations are presented in this section by using shooting technique. On this end, the formulated problem is converted into first order differential equations by making following assumptions:

18 $$ \left. \begin{gathered} f = z_{1} , \, f^{\prime} = z_{2} , \, f^{\prime\prime} = z_{3} ,f^{\prime\prime\prime} = z_{3}^{^{\prime}} , \, \hfill \\ \theta = z_{4} , \, \theta^{\prime} = z_{5} ,\theta^{\prime\prime} = z_{5}^{^{\prime}} , \, \hfill \\ \phi = z_{6} , \, \phi^{\prime} = z_{7} ,\phi^{\prime\prime} = z_{7}^{^{\prime}} , \hfill \\ \chi = z_{8} , \, \chi^{\prime} = z_{9} ,\chi^{\prime\prime} = z_{9}^{^{\prime}} . \hfill \\ \end{gathered} \right\} $$

In view of above assumptions, Eqs. (10–14) yield:

19 $$ z^{\prime}_{3} = \frac{{ - \left( {z_{1} z_{3} - (z_{2} )^{2} } \right)[1 + \left( {Wez_{3} } \right)^{1 - n} ] + M^{2} z_{2} \left[ {1 + \left( {Wez_{3} } \right)^{1 - n} } \right] + A\left( {z_{2} + \frac{\eta }{2}z_{3} } \right) - \lambda \left( {z_{4} - N_{r} z_{6} - R_{b} z_{8} } \right)}}{{\left( {1 + n\left( {Wez_{3} } \right)^{1 - n} } \right)}}, $$

20 $$ z^{\prime}_{5} = \frac{{\Pr \left( { - z_{1} z_{5} - A\frac{\eta }{2}z_{5} - N_{b} z_{5} z_{7} - N_{t} \left( {z_{5} } \right)^{2} - Q_{0} z_{4} } \right)}}{{\left( {1 + \frac{4}{3}Rd} \right)}}, $$

21 $$ z^{\prime}_{7} = - Scz_{1} z_{7} - \frac{{N_{t} }}{{N_{b} }}z_{7}^{\prime } - ScA\frac{\eta }{2}z_{7} + \sigma (1 + \delta z_{4} )^{{n^{ * } }} z_{6} \exp \left( { - \frac{{E_{1} }}{{1 + \delta z_{4} }}} \right), $$

22 $$ z^{\prime}_{9} = - Lbz_{1} z_{9} + Pe\left[ {z_{9} z_{7} + \left( {z_{8} + \Omega } \right)} \right] - LbA\frac{\eta }{2}z_{9} , $$

with boundary conditions:

23 $$ z_{1} \left( 0 \right) = 0,\, \, z_{2} \left( 0 \right) = 1,\, \, z_{5} \left( 0 \right) = - Bi\left[ {1 + z_{4} \left( 0 \right)} \right],\, \, N_{b} z_{7} \left( 0 \right) + N_{t} z_{5} \left( 0 \right) = 0,z_{8} \left( 0 \right) = 1, $$

24 $$ z_{2} \left( \infty \right) \to 0, \, z_{4} \left( \infty \right) \to 0, \, z_{6} \left( \infty \right) \to 0,\,z_{8} \left( \infty \right) \to 0. $$

## Validation of results

The validation of obtained numerical results via shooting technique has been verified in Table 1 by making comparison with analysis of Turkyilmazoglu^27^. A very excellent accuracy of results is observed between both studies. The grid independent is performed in Table 2 by considering different grid points for $\eta .$ It is noticed that an excellent solution accuracy has been observed when as number of grid points are increased.

**Table 1:** Solution comparison for $f^{\prime\prime}\left( 0 \right)$ with Turkyilmazoglu^27^ when $We = n = \lambda = A = 0.$

$M$	Turkyilmazoglu^27^	Present results
$0$	− 1.000000	− 1.000000
$0.5$	− 1.224744	− 1.224751
1	− 1.414213	− 1.414216
2.0	− 1.732050	− 1.732060

**Table 2:** Grid independent test for $$We = 0.2,$$ $$\lambda = 0.1,$$ $$N_{r} = 0.4,$$ $$R_{b} = 0.2,$$$$Pe = 0.1,$$$$M = 0.2,$$ $$N_{t} = 0.5,$$ $$N_{b} = 0.4,$$ $$Q_{0} = 0.2,$$$$A = 0.1,$$$$n = 0.4,$$ $$Sc = 0.2$$ and $$Lb = 0.1.$$

No. of grid points in $\eta$ direction with $\eta = 20$	$f^{\prime\prime}\left( 0 \right)$	$- \theta^{\prime}\left( 0 \right)$	$- \phi ^{\prime}\left( 0 \right)$	$- \chi^{\prime}\left( 0 \right)$
100	0.6853	0.4412	0.7045	0.8066
200	0.6855	0.4415	0.7048	0.8068
400	0.6855	0.4415	0.7048	0.8068
800	0.6855	0.4415	0.7048	0.8068

## Discussion of results

The transient two-dimensional magnetic flow of Cross nanofluid past a stretching sheet with convective boundary condition, thermal radiation, binary chemical reactions and heat source/sink is considered in the present model. The Buongiorno model incorporated into a currently developed framework for nanofluid. This section manifests the salient features and the rheological behaviour of various flow physical non-dimensional parameters associated in the dimensionless equations. Consideration is engrossed here to point out physical influence of these parameters on velocity, concentration, thermal and motile gyrotactic profiles graphically (Figs. 2, 3, 4, 5, 6, 7, 8, 9, 10, 11, 12, 13, 14, 15, 16, 17, 18, 19, 20, 21 and 22) by varying one parameter and other parameters are kept constant.

**Figure 2:**
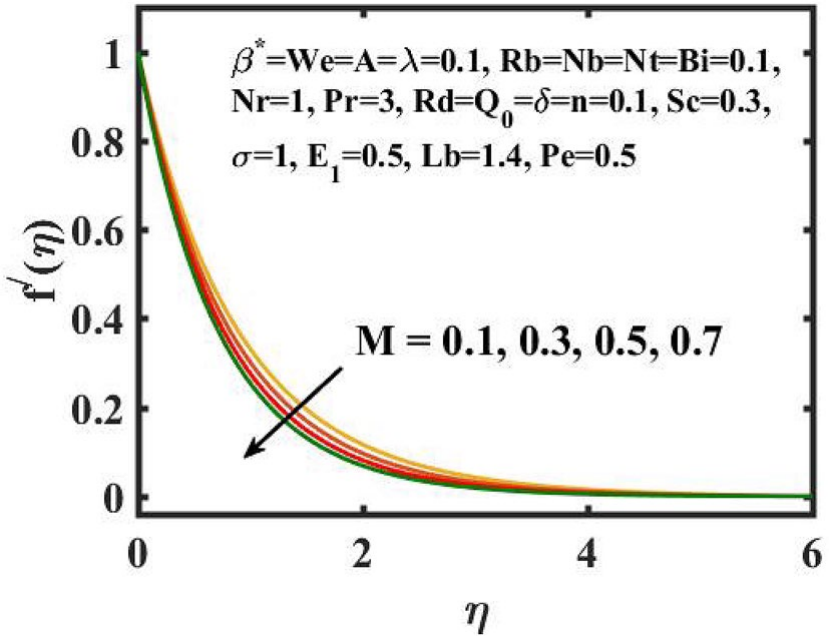
Encouragement of $M$ on $f^{\prime}(\eta )$.

**Figure 3:**
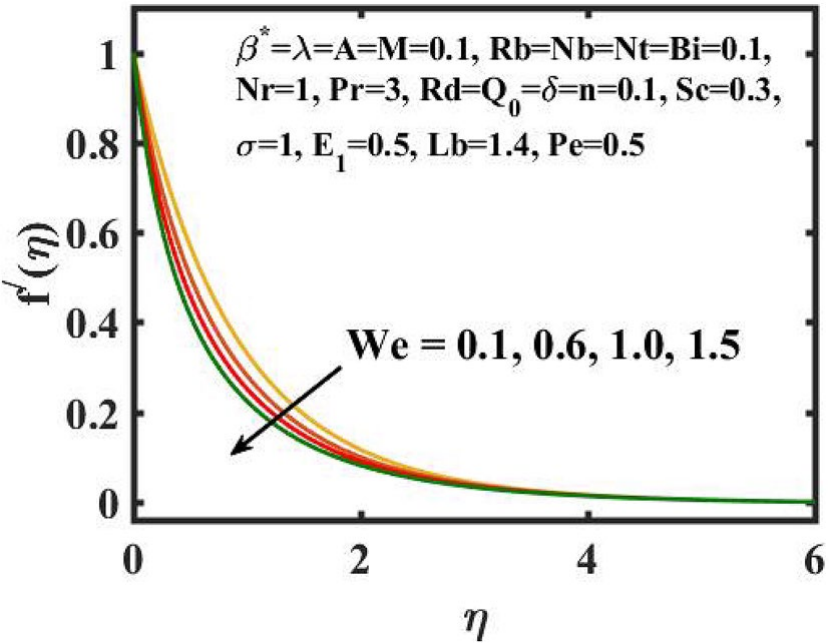
Encouragement of $We$ on $f^{\prime}(\eta )$.

**Figure 4:**
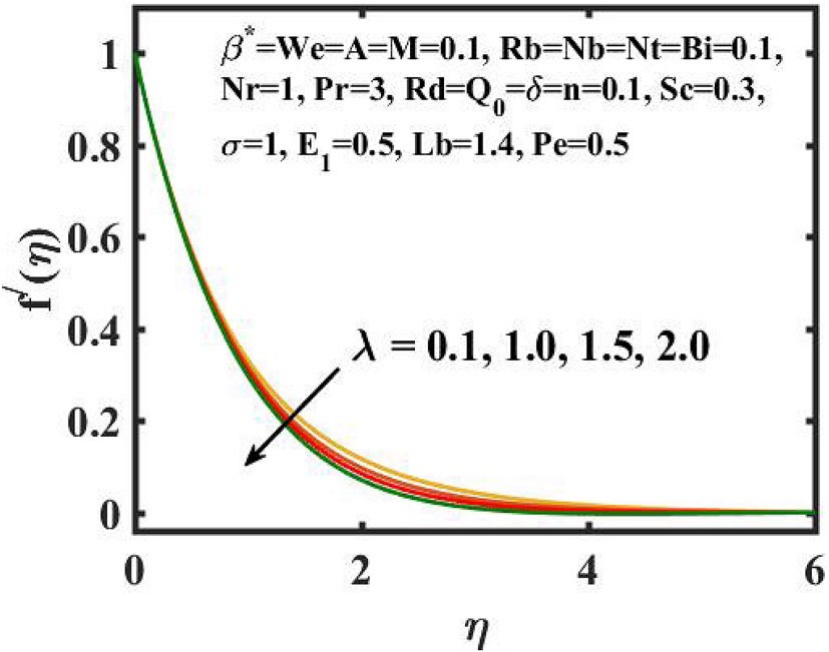
Encouragement of $\lambda$ on $f^{\prime}(\eta )$.

**Figure 5:**
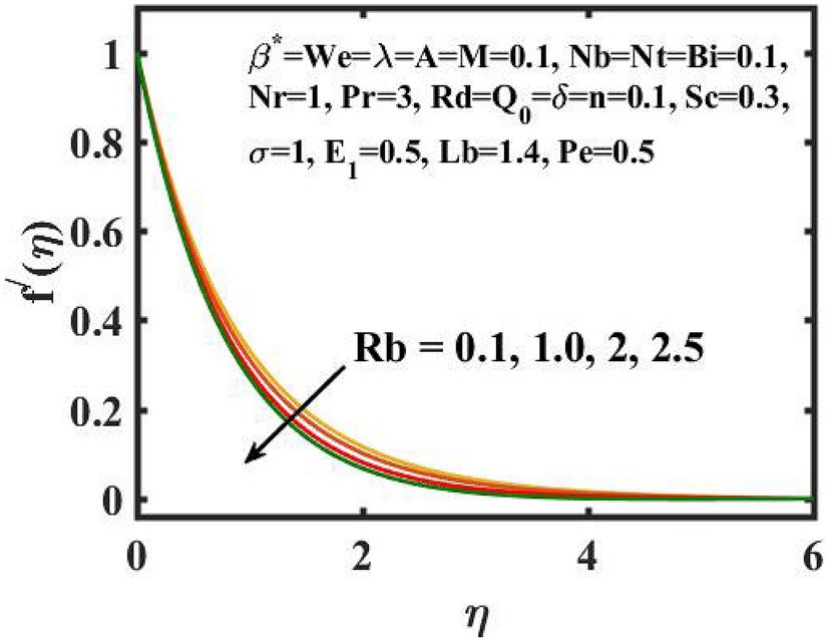
Encouragement of $Rb$ on $f^{\prime}(\eta )$.

**Figure 6:**
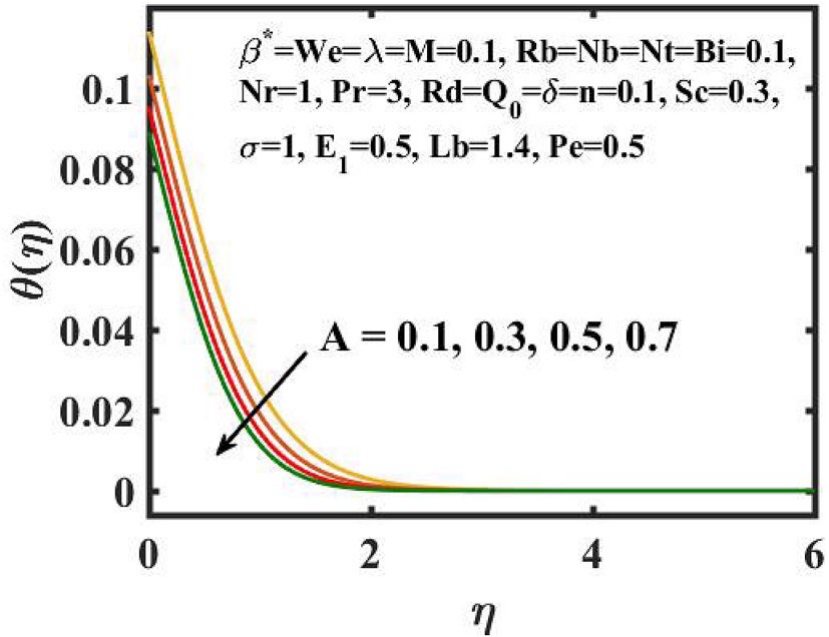
Encouragement of $A$ on $\theta (\eta )$.

**Figure 7:**
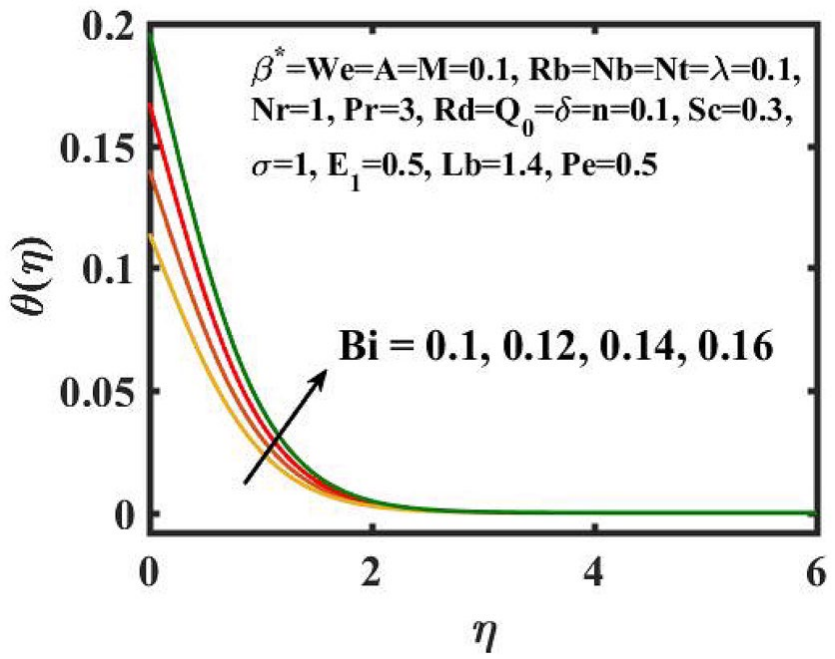
Encouragement of $Bi$ on $\theta (\eta )$.

**Figure 8:**
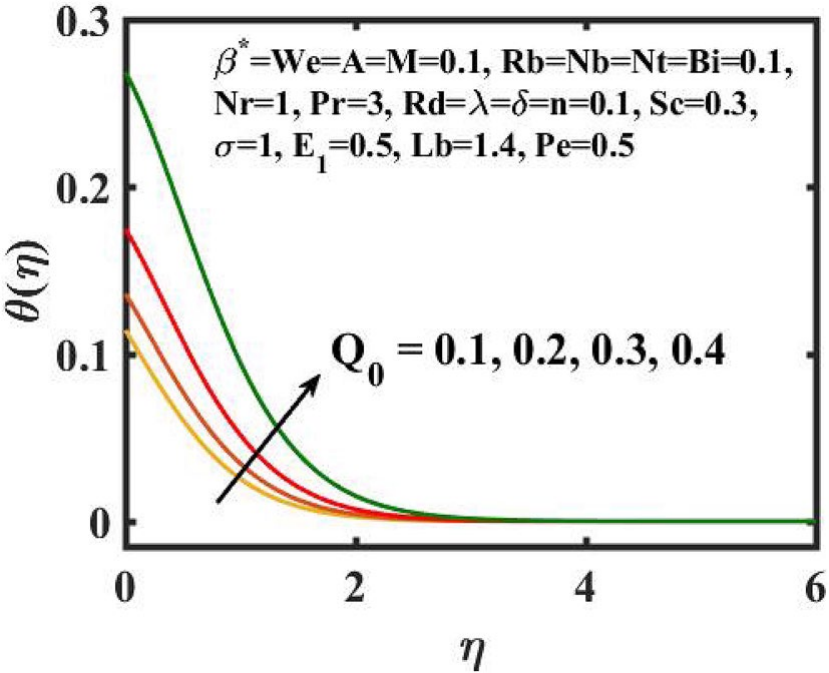
Encouragement of $Q_{0}$ on $\theta (\eta )$.

**Figure 9:**
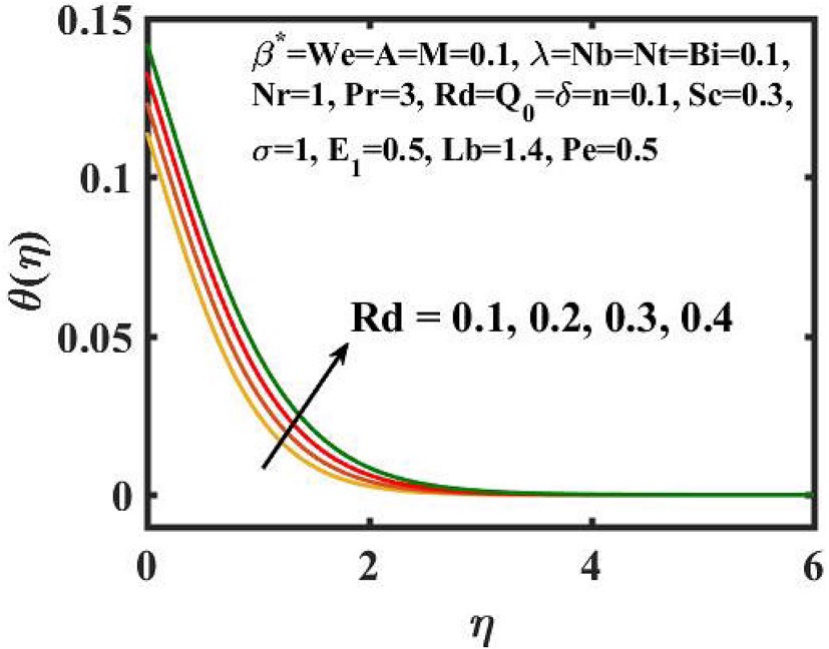
Encouragement of $Rd$ on $\theta (\eta )$.

**Figure 10:**
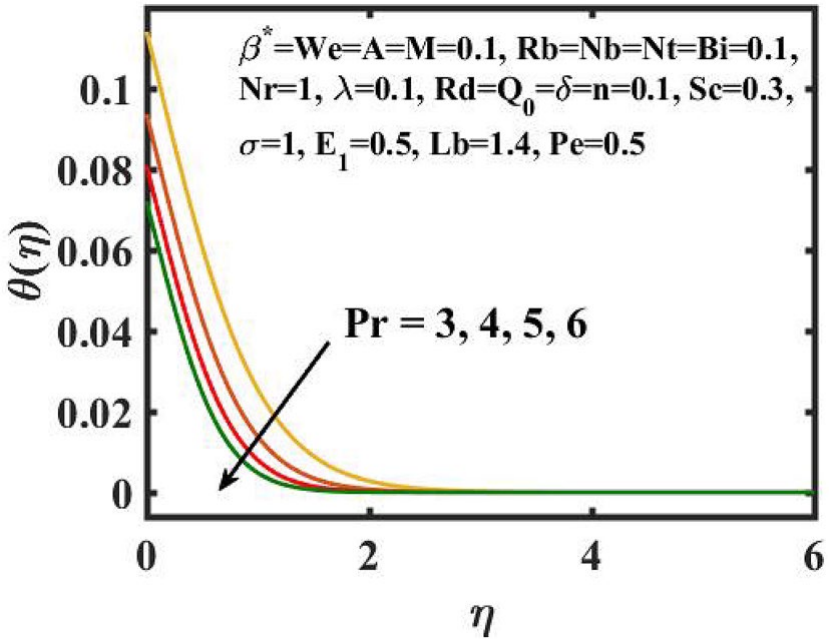
Encouragement of $\Pr$ on $\theta (\eta )$.

**Figure 11:**
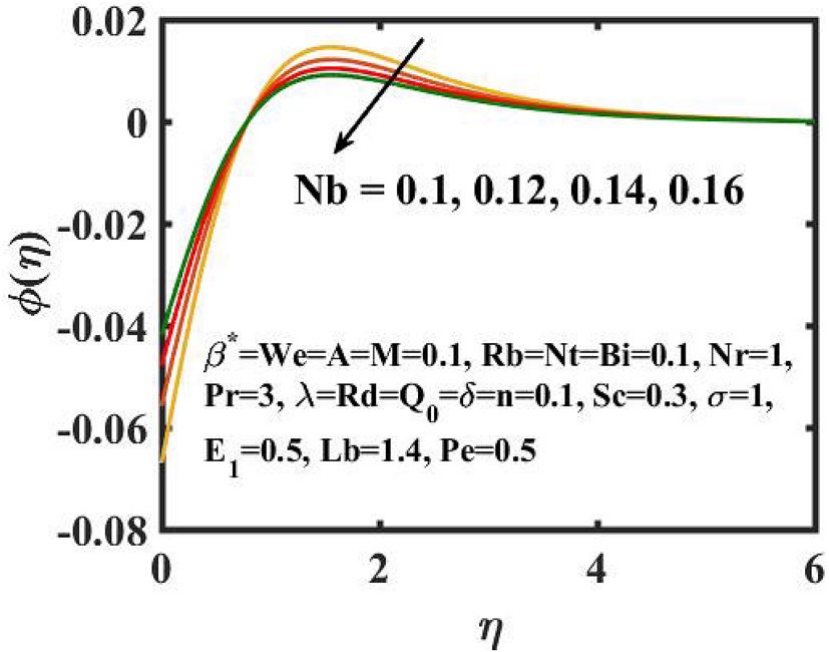
Encouragement of $Nb$ on $\phi \left( \eta \right)$.

**Figure 12:**
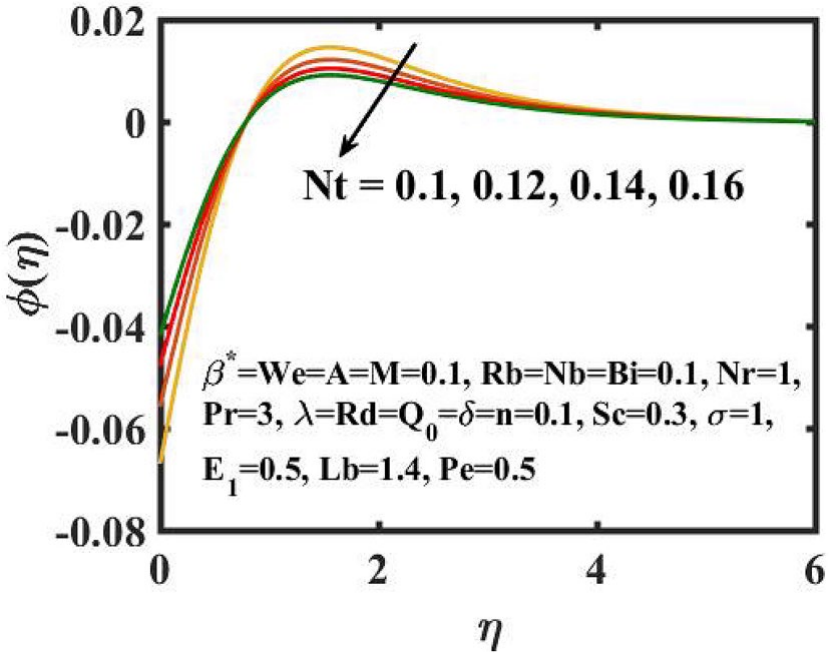
Encouragement of $Nt$ on $\phi \left( \eta \right)$.

**Figure 13:**
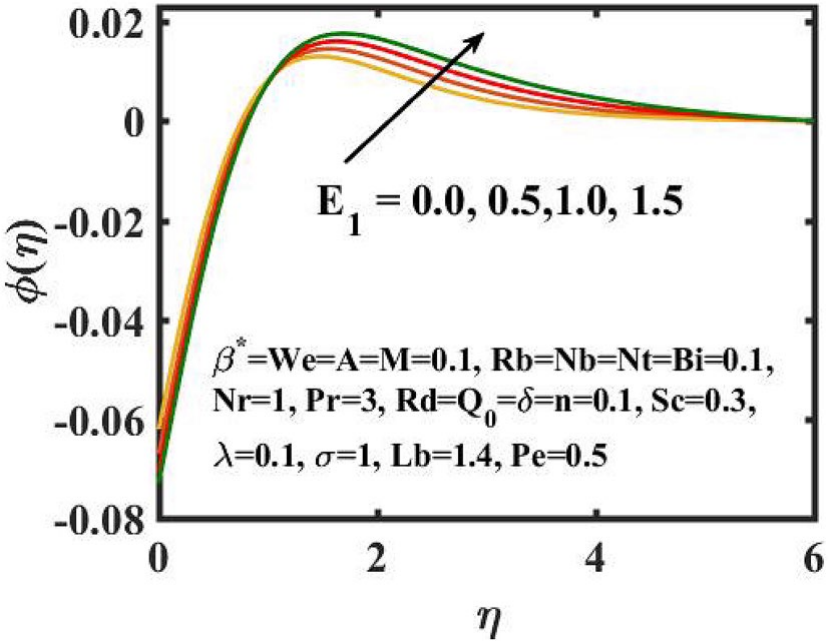
Encouragement of $E_{1}$ on $\phi \left( \eta \right)$.

**Figure 14:**
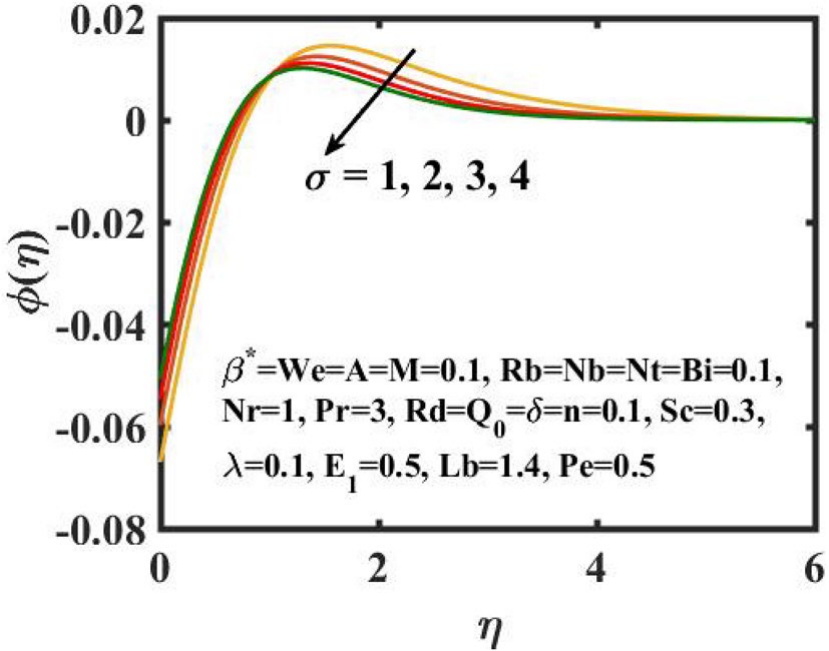
Encouragement of $\sigma$ on $\phi \left( \eta \right)$.

**Figure 15:**
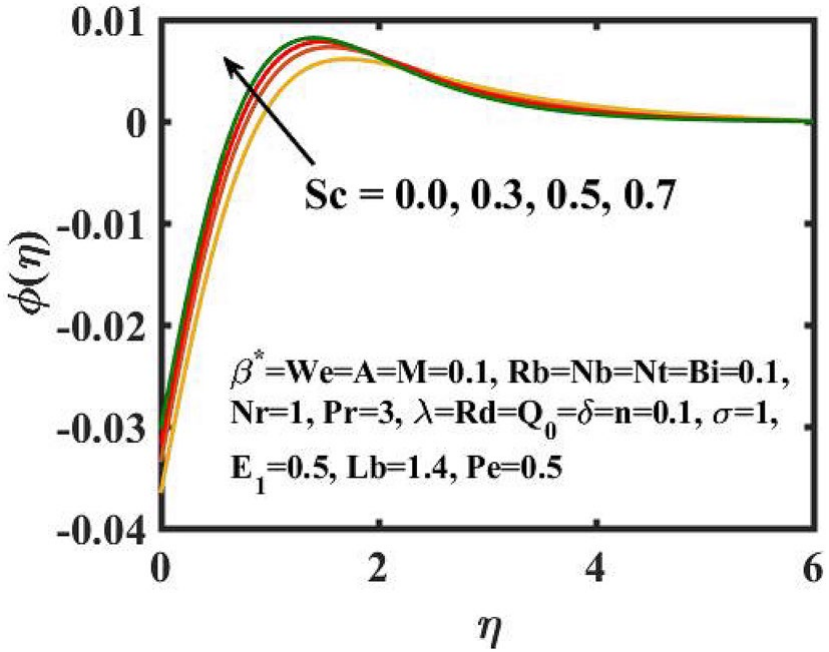
Encouragement of $Sc$ on $\phi \left( \eta \right)$.

**Figure 16:**
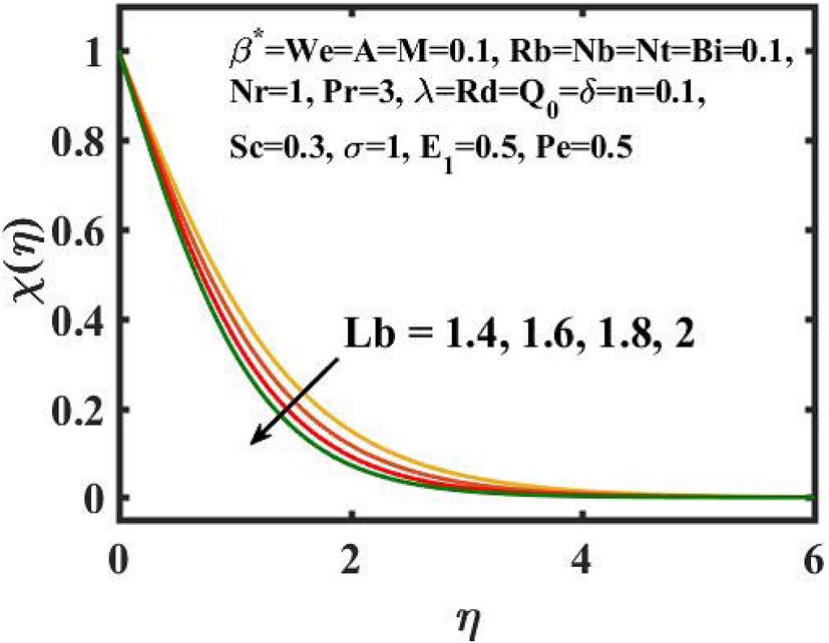
Encouragement of $Lb$ on $\chi \left( \eta \right).$

**Figure 17:**
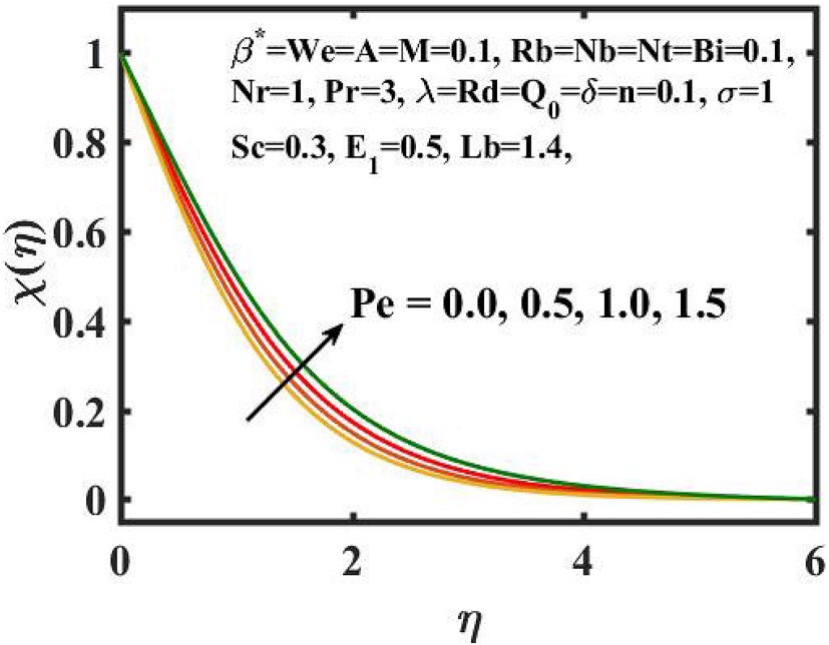
Encouragement of $Pe$ on $\chi \left( \eta \right).$

**Figure 18:**
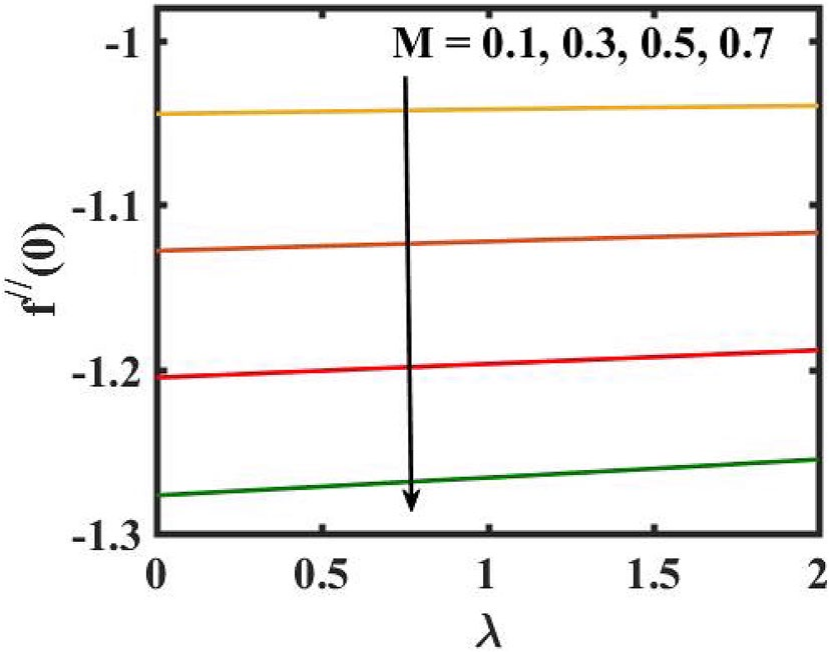
Encouragement of $M$ and $\lambda .$ on skin friction coefficient.

**Figure 19:**
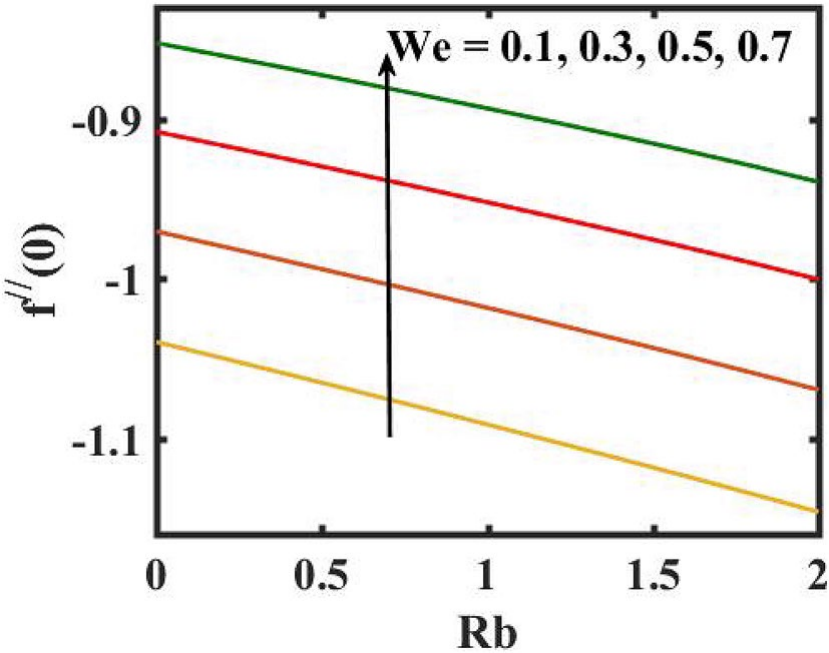
Encouragement $We$ and $Rb$ on skin friction coefficient.

**Figure 20:**
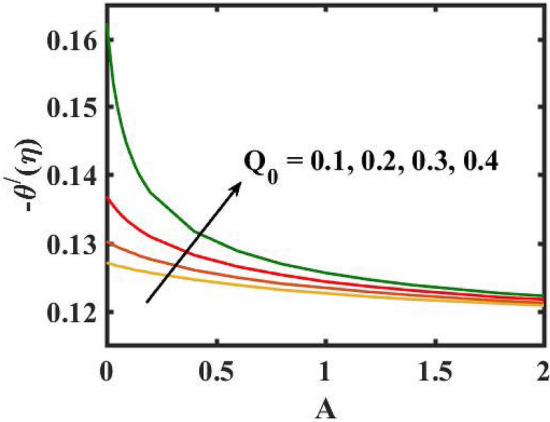
Encouragement of $Q_{0}$ and $A$ on local Nusselt number.

**Figure 21:**
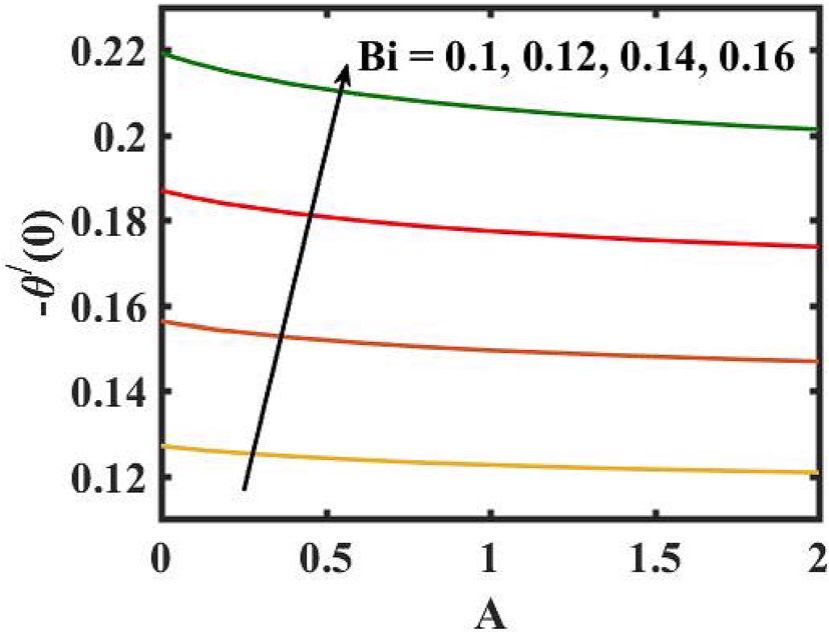
Encouragement of $Bi$ and $A$ local Nusselt number.

**Figure 22:**
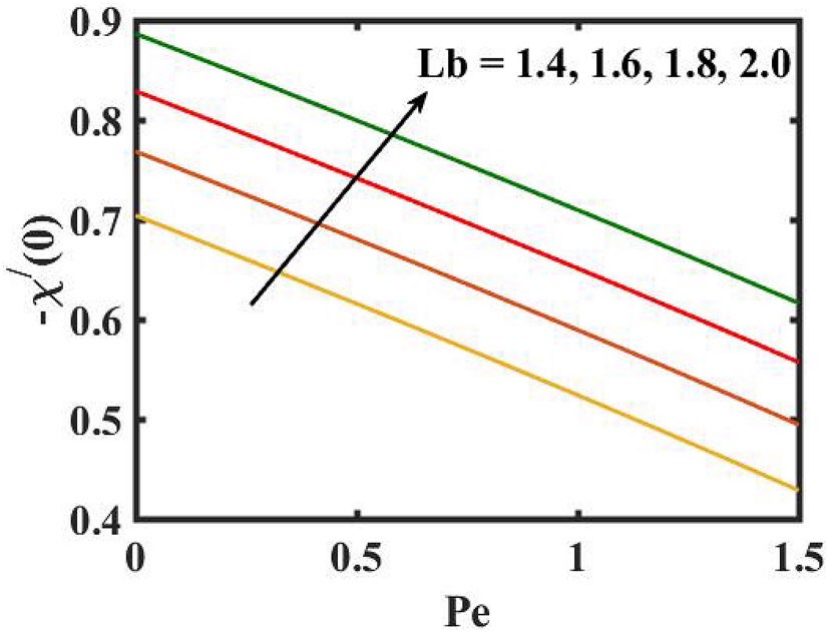
Encouragement of $Lb$ and $Pe$ on local density number of motile microorganisms.

Figure 2 depicts the flow control of the $M$ on the velocity profile. Here, increase in $M$ slowdowns the velocity and momentum of the fluid. Practically, it has been noticed that the existence of a magnetic field in the stream field region decelerates the fluid motion. These findings suggest that magnetization force provides additional struggle to the stream which declines its velocity. Figure 3 demonstrates the influence of the $We$ on velocity gradient. Here, it is observed that the rise in $We$ turn down the fluid particles motion slowly. Here, upsurge in material relaxation time affords additional struggle to fluid stream which diminishes the velocity gradient. Figure 4 is devoted to scrutinize the estimation in the velocity profile for several values of $\lambda$. Physically, by increasing $\lambda$, the inertial force is overcome by the buoyancy force, which results in declination of the velocity gradient. Figure 5 is engrossed to depict the levering of $Rb$ on the velocity gradient. Outcome of the figure reveals that the cumulative values of $Rb$ deteriorates the velocity gradient. This deteriorating behaviour is caused by the fact that $Rb$ is related to the buoyancy force caused by bioconvection, which allows the velocity profile to decay.

Figure 6 is displayed to envisage the domination of $A$ on the thermal profile. The inclination in $A$ slowdowns the heat transference. Figure 7 manifests the characteristic of $Bi$ on thermal gradient. Here, the increased values of $Bi$ increase the heat transference. Physically describes that even more heating is delivered from the surface to the nanoparticles, resulting in an increase in the temperature gradient. Figure 8 demonstrates the behaviour of $\;Q_{0}$ on temperature profile. The larger $\;Q_{0}$ enhances the thermal profile. In fact, Internal heat generation/absorption either improves or dampens the heat transport. A incremental increase in $\;Q_{0}$ increases thermal gradient, showing mechanically that an increase in heat source intensity contributes to a greater thermal diffusion layer, which may increase thermal gradient. Figure 9 highlights the outcome of the $Rd$ on thermal profile. The increase in radiation parameter upsurges the thermal distribution. Here, more heat is applied to active liquid due to upshot in radiation phenomenon. As a result, heat transfer escalates.

Figure 10 describes the behaviour of the $\Pr$ on thermal profile. The inclination in $\Pr$ declines the thermal gradient.Lower $\Pr$ equate to higher thermal diffusivity, although higher values result in higher diffusivity. As a consequence of this justification, the temperature is reduced. Shear thickening activity is characterised by gradual thinning of the boundary layer. Furthermore, by definition, the $\Pr$ is inversely related to thermal diffusivity, resulting in a decrease in the thermal profile.

The graphical results for mass transference for varied values of the $Nb$ is expressed in Fig. 11. Here, mass distribution diminishes for inclined $Nb$.Concentration gradient decays after more particles are pushed in the reverse way of the solutal distribution to preserve solution homogeneity. Figure 12 determines the sway of $Nt$ over concentration gradient. The escalation in $Nt$ declines the mass transfer. Physically, raising in the $Nt$ causes an increase in thermophoretic energy, which raises the fluid temperature as well as the nanoparticle concentration due to the migration of nanoparticles from warm to chill regions. Figure 13 exhibits the feature of $E_{1}$ on concentration distribution. Larger values of $E_{1}$ enhances the concentration field. As the $E_{1}$ becomes larger, the modified Arrhenius mechanism decays. This eventually inspires the procreative chemical reaction, which causes the concentration of nanoparticles to increase. Figure 14 exemplifies the fluctuation of the concentration field with $\sigma$. The concentration of species in the boundary layer decreases as the value of the $\sigma$ increases. This is because the chemical reaction in this method consumes the chemicals, resulting in a reduction in the concentration profile. Variation in mass transference with respect to $Sc$ is portrayed in Fig. 15. Mathematically, a dimensionless number relating mass diffusivity with momentum diffusivity yielding a fluid flow is treated as Schmidt number. These two terms are physically called as the hydrodynamic thickness layer and mass transport layer. The maximum concentration of nanoparticles corresponds to the smallest $Sc$.

**Figure 23:**
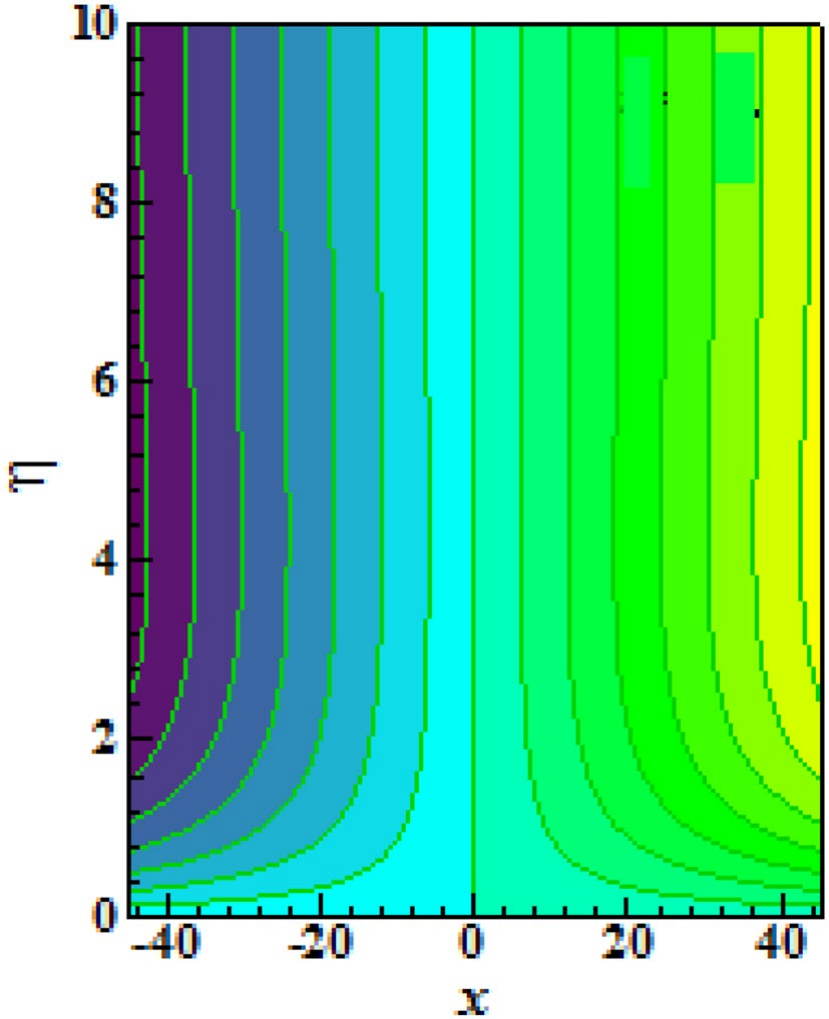
Streamlines for flow parameters when $We = 0.2,$ $\lambda = 0.1,$ $N_{r} = 0.4,$ $R_{b} = 0.2,$ $Pe = 0.1,$ $M = 0.2,$ $N_{t} = 0.5,$ $N_{b} = 0.4,$ $Q_{0} = 0.2,$ $A = 0.1,$ $n = 0.4,$ $Sc = 0.2$ and $Lb = 0.1.$

**Figure 24:**
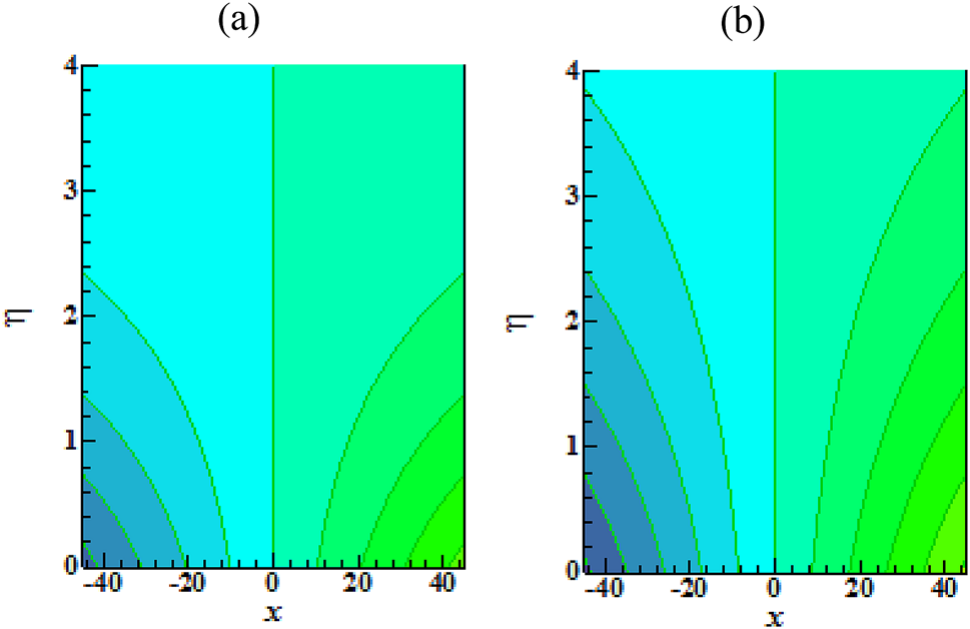
Contour plots of isotherms for when (**a**) $Ha = 0.1$ and (**b**) $Ha = 0.5.$

Figure 16 elucidates the depreciation in the motile microorganism distribution for various values of the $Lb$. With enlarging value of $Lb$, a perceptive motile microorganism profile is observed. Figure 17 demonstrates the impact of the $Pe$ on microorganism profile. A rise in $Pe$ results in a poorer motile microorganism profile. Increased Pe induces a reduction in the diffusivity of microorganisms and hence a decrease in the motile density of fluid. Figure 18 displays the variation of skin friction coefficient against $M$ and $\lambda .$ Here, escalating values of $M$ declines the coefficient of skin friction. Figure 19 shows the skin friction coefficient variation against $We$ and $Rb.$ Here, escalating values of $We$ improves the coefficient of skin friction. The variation of local Nusselt number against $Q_{0}$ and $A$ is demonstrated in Fig. 20. Here, local Nusselt number upsurges for growing values of $Q_{0}$ and declines for escalating values of $A$. The change in local Nusselt number against $Bi$ and $A$ is established in Fig. 21. Here, local Nusselt number acts as a growing function of $Bi$ and declining function of $A$. The change in local density number of motile microorganisms against $Lb$ and Pe is established in Fig. 22. Here, density number of motile microorganisms increases for growing values of $Lb$ and declines for rise in values of $Pe.$ Figures 23 and 24 are sketched for stream functions subject to various flow parameters^28^.

## Conclusions

The transient magnetic flow of Cross nanofluid past a stretching sheet with convective boundary condition, thermal radiation effects, binary chemical reactions and heat source/sink is considered in the present model. The effect of Brownian motion and thermophoresis is incorporated into a currently developed framework for nanofluid. The salient features and the rheological behaviour of various flow physical parameters on velocity, concentration, thermal and motile gyrotactic profile are deliberated graphically. The key conclusions of the current study are:

- The escalation in $M$ and $Rb$ slowdowns the velocity and momentum of the fluid.
- The rise in $We$ turn down the fluid particles motion slowly.
- The increased values of $Bi$ and $Q_{0}$ intensify the heat transference.
- The increase in radiation parameter upsurges the thermal boundary but, converse trend is seen for escalating Prandtl number.
- The escalation in $Nt\,{\text{and}}\,Nb$ declines the mass transfer.
- An augmentation in the $Pe\,{\text{and}}\,Lb$ deteriorates themicroorganism profile.
- The local Nusselt number acts as a growing function of $Q_{0} \,{\text{and}}\,Bi$ and declining function of $A$.
- The density number of motile microorganisms acts as a growing function of $Lb$ and declining function of $Pe.$

## Data Availability

The data that support the findings of this study are available within the article, the data are made by the authors themselves and do not involve references of others.
